# Studying the Effect of Hepatic Steatosis on Liver Stiffness Measurement (LSM) in HBV Patients With and Without Therapy

**DOI:** 10.1155/ijh/5574421

**Published:** 2025-10-28

**Authors:** Elena Lak, Farzad Jasemi Zergani, Zahra Shokati Eshkiki, Eskandar Hajiani, Valiollah Talebi, Samira Mohamadi

**Affiliations:** Alimentary Tract Research Center, Clinical Sciences Research Institute, Ahvaz Jundishapur University of Medical Sciences, Ahvaz, Iran

**Keywords:** hepatic fibrosis, hepatic steatosis, hepatitis, LSM index, transient elastography

## Abstract

**Introduction:**

The issue of fatty liver disease is becoming more widely acknowledged as a global health concern. This disorder manifests as inflammation brought on by varied degrees of fat buildup in the liver tissue. Since studies have shown that fatty liver is a major factor affecting the prognosis of patients with chronic hepatitis, the coexistence of viral hepatitis and fatty liver is noteworthy. Transient elastography is a useful technique for identifying and measuring the degree of steatosis. The liver stiffness measurement (LSM) index might be a good substitute for these patients if it shows a high diagnostic value in identifying hepatic steatosis.

**Methods:**

This pilot study presents an analytical cross-sectional analysis of 53 hepatitis B patients (26 men and 27 women) who sought care at Aria Hospital in Ahvaz, Iran, between April 2023 and November 2024. Participants were divided into two groups: 34 patients in the treatment group and 19 patients in the nontreatment group. The subjects' prevalence of hepatic fibrosis was evaluated using transient elastography and the LSM index. Further comparisons between treatment-receiving and nonreceiving patients were conducted using LSM.

**Results:**

The prevalence of steatosis was found to be 25% in the untreated group and 26.7% in the treatment group. In the treatment group, the incidence of fibrosis was 58.8%, while in the untreated group, it was 57.9%. In both the treatment-treated and nontreated groups of hepatitis B patients, the associations between the study variables and the LSM index were evaluated. Every correlation that was found was not statistically significant. Additionally, the LSM's diagnostic value for hepatic steatosis was evaluated. In the treatment group, the test showed limited diagnostic value, while in the untreated group, it showed satisfactory diagnostic value.

**Conclusions:**

In patients with hepatitis B, the LSM derived from transient elastography does not show a statistically significant correlation with factors such as age, gender, body mass index (BMI), or degree of hepatic steatosis. According to this study, the LSM number is not a reliable indicator for identifying hepatic steatosis in patients with hepatitis B when compared to the CAP score (AUC = 0.69 and 0.74).

## 1. Introduction

The importance of fatty liver disease (FLD) as a global health concern is becoming more widely acknowledged [1]. Different degrees of fat buildup in the hepatic cells and tissue are reflected in the condition, which causes inflammation that can progress to cirrhosis, fibrosis, and finally liver failure [2]. As the number of cases of diabetes, obesity, and metabolic syndrome has increased, so too has the incidence of this disease [3]. FLD has surpassed infectious diseases as the most common chronic liver disease as a result of the general improvement in human living standards and health conditions in communities [3]. The most common technique for determining the degree of liver fibrosis in patients with fatty livers is biopsy [4]. However, there are unique risks associated with this invasive procedure [2]. Scientists have wanted to develop a low-risk, noninvasive diagnostic technique for assessing liver fibrosis. More people have been diagnosed with liver enzyme disorders as a result of increased physician requests for liver function tests (LFTs) and easier access [5]. The presence of FLD in these individuals is usually confirmed by subsequent diagnostic procedures [6]. Numerous risk factors, such as alcohol use, metabolic disorders, certain medications, and nutritional deficiencies, are linked to fatty liver [3, 7].

Steatosis and cirrhosis are just two of the many liver disorders that fall under the umbrella of non-alcoholic fatty liver disease (NAFLD). These conditions have the potential to develop into liver cancers and cause death if they are not properly monitored and treated [8]. NAFLD is linked to a higher risk of dying from cardiovascular diseases and usually develops in the setting of metabolic syndrome disorders, such as obesity, insulin resistance, hypertension, and dyslipidemia [9]. According to numerous studies, the prevalence of NAFLD varies between 5% and 20% in the general population and can surpass 40% in patients with Type 2 diabetes and those who are obese [3]. About 25% of FLD patients develop cirrhosis, and 7% of these patients develop end-stage liver disease (ESLD), which requires a liver transplant [10]. Studies have assessed transient elastography, more especially vibration-controlled transient elastography (VCTE), as a useful technique for measuring liver stiffness and determining the degree of liver fibrosis [11]. Several confounding factors have been found to influence the liver stiffness measurement (LSM) index, such as alcohol use, serum ALT, total bilirubin, extrahepatic cholestasis, congestive heart failure, and active hepatitis with parenchymal liver damage [12]. However, other research has indicated that the LSM index is unaffected by the degree and presence of hepatic steatosis [13]. LSM significantly decreased in patients with hepatic steatosis, according to a different study looking into NAFLD patients [14]. Due to inconsistent findings from earlier research, the impact of hepatic steatosis on the LSM index is still up for debate. Additionally, outcomes vary depending on the severity of steatosis and the etiology of the disease. On the other hand, the impact of hepatic steatosis on the LSM index may change depending on when treatment for hepatitis B patients begins [15]. As mentioned, for patients with hepatitis B, the LSM index may provide a useful diagnostic tool for liver fibrosis and a noninvasive, affordable substitute. Given the conflicting results regarding the impact of hepatic steatosis on the LSM index and the paucity of research addressing this relationship in the context of treatment status, the purpose of this study is to examine the effect of hepatic steatosis on the LSM index in patients with HBV, taking into account both treated and untreated groups.

## 2. Materials and Methods

### 2.1. Study Design

For this cross-sectional pilot study, 53 individuals who were diagnosed with hepatitis B participated. There were a total of 27 women and 26 men who were referred to the Gastroenterology Department of Aria Hospital in Ahvaz between April 2023 and November 2024 and were included in the study (Ethics Committee Number: IR.AJUMS.HGOLESTAN.REC. 1402.081). All study participants provided their informed consent. Two groups were formed: 34 patients who were receiving therapy and 19 patients who were not receiving treatment. According to current recommendations for treating HBV, patients receive tenofovir by mouth as either tenofovir disoproxil fumarate (TDF) 300 mg once a day or tenofovir alafenamide (TAF) 25 mg once a day. Therapy continued until there was proof that the hepatitis B surface antigen (HBsAg) had been lost, which was verified by two negative tests done at least 6 months apart. The length of treatment varied from patient to patient depending on how well their viruses responded.

Inclusion requirements are as follows: (1) A diagnosis of hepatitis B has been confirmed. (2) There was no detection of any other kind of hepatitis that was confirmed. (3) An age requirement of 18 years is required. (4) No reliance on alcohol is present. (5) In addition to the controlled attenuation parameter (CAP) value, the patient's medical records include the necessary demographic and laboratory data on the patient. (6) Lack of an HIV infection verified diagnosis. The absence of any final stages of liver illness or a verified diagnosis of hepatic cancer. The body mass index (BMI) is below 35, and the serum ALT levels are higher than 200 IU/L.

Through the use of transient elastography, the LSM index was utilized to assess the frequency of liver fibrosis. This was accomplished by taking liver stiffness measures for each patient utilizing a 3.5 MHz probe.

After determining the treatment status of the patients, the LSM index's evaluation of the frequency of liver fibrosis was then determined and matched. After that, the medical records of the patients produced data, which included demographic information, laboratory results, and the first findings associated with the fibrosis scan.

### 2.2. Statistical Analysis

The mean and standard deviation (SD) were used to exhibit numerical data, while frequency and percentage were used to display categorical variables. In contrast, a parametric independent *t*-test was used when the distribution had a Gaussian pattern. In contrast, a Mann–Whitney *U* test was used for inference and comparative analysis if the distribution had not been Gaussian. The chi-square test or, where applicable, Fisher's exact test was used for categorical variables. For all statistical tests, a *p* value of less than 0.05 was deemed significant. We used the ROC (receiver operating characteristic) curve assessment to evaluate the diagnostic efficacy of LSM in spotting hepatic steatosis among patients with hepatitis B, which was evaluated independently of treatment status.

## 3. Results

There were no dropouts during the study. Results from the independent variables are presented in Table 1.

The study recorded a complete retention of participants, with no instances of dropout observed. This table presents the outcomes derived from the independent variables:

Hepatic steatosis was classified as S1, which corresponds to a range greater than 5% and up to 33%, while S0 is defined as 5% or less. The occurrence of hepatic steatosis in the overall patient population was 26.6%. In the subset of patients not receiving treatment, the rate was 25%, while in the treatment group, it was 27.6%. The analysis revealed no statistically significant difference in the prevalence of hepatic steatosis between the two groups (*p* = 0.54).

Liver fibrosis is classified as F1 when the LSM falls within the range of 7 and 8.2 kPa, with F0 defined as an LSM of less than 7 kPa. The occurrence of liver fibrosis in the overall patient population was 58.5%. In the subgroup of patients not receiving treatment, the rate was 57.9%, while in those undergoing treatment, it was 58.8%. The analysis revealed no statistically significant difference in the prevalence of liver fibrosis between the two groups (*p* = 0.47).

The treatment group exhibited a weak inverse correlation between BMI and LSM index, which did not reach statistical significance (correlation coefficient = −0.22 and *p* = 0.20). Furthermore, within the cohort of untreated patients, a weak correlation was identified, although it did not reach statistical significance (correlation coefficient = 0.12 and *p* = 0.60).

The measurement of hepatic steatosis was conducted utilizing the CAP value. A weak correlation was noted between hepatic steatosis and the LSM index in both groups, regardless of treatment, with correlation coefficients of 0.22 and 0.33, respectively. These values were not statistically significant in either group (*p* = 0.25 and 0.07) (Table 2).

A weak correlation was noted between age and the LSM index in both patient groups, regardless of treatment status, with correlation coefficients of 0.09 and 0.05, respectively. These values were not statistically significant in either group (*p* = 0.41 and 0.72).

As shown in Table 2, the treatment group exhibited a weak inverse correlation between gender and the LSM index, which did not reach statistical significance (correlation coefficient = −0.21 and *p* = 0.14). In the untreated group, a weak correlation was noted, which did not reach statistical significance (correlation coefficient = 0.11 and *p* = 0.56).

Using ROC curve analysis, the diagnostic efficacy of LSM in spotting hepatic steatosis among patients with hepatitis B was evaluated independently of treatment status. The criterion for hepatic steatosis diagnosis was based on the CAP level; thus, the threshold of 238 dB/m or above was defined (Table 3).

With an area under the curve (AUC) of 0.69, the group of patients undergoing treatment showed a weak outcome; nevertheless, this outcome did not reach statistical significance (*p* = 0.09). This patient cohort had an LSM threshold of 8.15 kPa, showing a sensitivity of 0.75 and a specificity of 0.62. An LSM threshold of 6.6 kPa showed a sensitivity of 1 and a specificity of 0.48 by contrast.

The AUC for the treated patient group was 0.74, which is regarded as a reasonable outcome even though this result did not reach statistical significance (*p* = 0.13). This patient group revealed an LSM threshold of 8.05 kPa with a sensitivity of 0.75 and a specificity of 0.66. By contrast, an LSM threshold of 6.75 kPa revealed a sensitivity of 1 and a specificity of 0.66.

## 4. Discussion

For this study, patients were divided into two groups: 34 were receiving treatment, and 19 were not receiving treatment. Following this, the prevalence of hepatic steatosis and fibrosis was evaluated in both groups of hepatitis B patients, those who were receiving treatment and those who were not receiving treatment. In the group that received treatment, the incidence of hepatic steatosis was 27.6%, while in the group that did not receive treatment, it was 25%. In the group that received therapy, the prevalence of liver fibrosis was 58.8%, whereas in the group that did not receive treatment, it was 57.9%. There were no statistically significant differences seen between the two groups.

The correlation between variables such as hepatic steatosis, age, gender, and BMI and the LSM index was evaluated using correlation tests in both treated and untreated hepatitis B patient groups. The results of these tests indicated that there were no statistically significant correlations between these variables and the LSM index in hepatitis B patients.

Using the ROC curve, the researchers investigated the diagnostic efficacy of the LSM index for hepatic steatosis in both treated and untreated cohorts. The results showed that the LSM index had a low diagnostic value (AUC = 0.69) in the treated hepatitis B patient group, while the untreated patient group had an acceptable diagnostic value (AUC = 0.74). Despite this, none of these findings achieved the level of statistical significance.

Previous research [16–18] showed that the use of antiviral medicine in individuals with hepatitis B may be associated with the reversal of liver fibrosis and a decrease in the LSM index during treatments. Furthermore, in that study, increased levels of AST and ALT were shown to correspond with a more substantial decline in the LSM score. This conclusion was also supported by the present investigation, which validated the association between the two variables. In the study that was conducted in 2008 by Oliveri and colleagues, it was shown that serum ALT levels, as opposed to serum AST levels, are the ones that connect with the LSM index and the prediction of liver fibrosis [19]. To identify liver fibrosis, the study determined that a cutoff of 7.5 kPa is appropriate, whereas the cutoff for diagnosing liver cirrhosis is 11.8 kPa [19].

There are also differences of opinion among the many studies that have been conducted on the relationship between the severity of hepatic steatosis and higher levels of liver steatosis in those who have hepatitis B. Even though our analysis did not uncover a statistically significant connection between the severity of liver fibrosis and the amount of hepatic steatosis in patients, Jie et al. found that there is a direct connection between the two. They discovered that hepatic steatosis is a factor that may be used to predict the LSM index independently. The presence of moderate to severe steatosis, as opposed to mild steatosis, constitutes a considerable increase in the likelihood of overestimating the LSM index in patients who are infected with hepatitis B [20–22].

There is no significant link between the LSM index and the patient's age, gender, BMI, or the degree of hepatic steatosis of the patient. When it comes to determining whether or not a person has hepatitis B, the LSM index that is obtained from transient elastography is not a suitable replacement for the CAP.

Because of this study, our understanding of the relationship between hepatitis B treatment, liver fibrosis, and other clinical markers that are connected to these conditions has been improved. However, doing research with a larger sample size and over a longer period may make it easier to have a more comprehensive understanding of the impact that hepatitis B treatment has on the stiffness of the liver.

There is no statistically significant connection between the LSM index of transient elastography and any of the factors that were examined, including age, gender, BMI, and the degree of hepatic steatosis, according to the data. Furthermore, in contrast to the CAP index of the FibroScan, the LSM is not an accurate marker for diagnosing hepatic steatosis in people who have hepatitis B. This is because the LSM does not measure the thickness of the liver.

## 5. Limitations/Future Work

The main limitation of this study is the small participant pool. Detecting small to moderate variations in LSM can be challenging. The inability to achieve these targets is due to the constraint of recruiting individuals from a singular location and the limited duration assigned for the study. This suggests that the potential for a Type II error cannot be overlooked in the context of smaller effect sizes. The data suggest the need for further multicenter trials to validate these findings.

## 6. Conclusion

In this pilot study, transient elastography and the LSM index were used to determine the extent to which the participants exhibited hepatic fibrosis. Additional comparisons between patients who received therapy and those who did not receive treatment were carried out with the help of LSM. When it comes to individuals who have hepatitis B, the LSM that is determined from transient elastography does not exhibit a statistically significant link with parameters such as age, gender, BMI, or the degree of hepatic steatosis. The results of this research indicate that the LSM number is not a valid indication for diagnosing hepatic steatosis in individuals who have hepatitis B. This is in contrast to the CAP score, which has an AUC of 0.69 versus 0.74.

## Figures and Tables

**Table 1:** Independent variables in hepatitis B patients with and without treatment.

		**Age**	**BMI**	**AST**	**ALT**	**ALK-P**	**FBS**	**CAP**	**LSM**
Without treatment	Number	19	19	19	19	19	19	16	19
	Mean	49.68	36.04	30.63	28.94	193.47	98.01	167.56	7.74
	SD	14.94	3.39	18.29	17.91	62.52	22.64	19.92	2.56
	Max	76	33	76	90	338	130	205	13.60
	Min	29	21.2	9	7	111	10.30	151	4.40

Treatment	Number	34	34	34	34	34	34	29	34
	Mean	46.26	24.37	28.55	34.73	171.94	100.41	173.68	9.40
	SD	12.43	3.34	7.50	14.17	35.30	9.50	33.02	4.94
	Max	72	33	51	84	263	121	260	26.50
	Min	24	18	16	13	112	79	107	4.10

Total	Number	53	53	53	53	53	53	45	53
	Mean	47.49	24.96	29.30	32.66	179.66	99.55	171.51	8.81
	SD	13.34	3.36	12.35	15.69	47.46	15.36	28.94	4.29
	Max	76	33	76	90	338	130	260	26.50
	Min	24	18	9	7	111	10.30	107	4.10
	*p* value	0.77	0.08	0.56	0.20	0.17	0.59	0.50	0.17

**Table 2:** Correlations between study variables and LSM index.

**Variables**	**LSM**	**Correlation**	**p** **value**
Hepatic steatosis	Treatment	0.22	0.25
	Without treatment	0.33	0.07
Age	Treatment	0.09	0.41
	Without treatment	0.05	0.72
Gender	Treatment	−0.21	0.14
	Without treatment	0.11	0.56
BMI	Treatment	−0.22	0.20
	Without treatment	0.12	0.60

**Table 3:** Diagnostic value of LSM for hepatic steatosis in hepatitis B patients.

**Indexes**	**Patients with treatment**	**Patients without treatment**
AUC	0.69	0.74
*p* value	0.09	0.13
Cutoff 1	8.15 kPa	8.05 kPa
Cutoff 2	6.6 kPa	6.75 kPa

## Data Availability

The full anonymized individual patient dataset is available upon request to the corresponding authors.
